# Evolutionary epidemiology models to predict the dynamics of antibiotic resistance

**DOI:** 10.1111/eva.12753

**Published:** 2019-01-21

**Authors:** François Blanquart

**Affiliations:** ^1^ Centre for Interdisciplinary Research in Biology (CIRB), Collège de France, CNRS, INSERM PSL Research University Paris France; ^2^ IAME, UMR 1137, INSERM Université Paris Diderot Paris France

**Keywords:** adaptation, antimicrobial resistance, bacterial genomics, mathematical modelling

## Abstract

The evolution of resistance to antibiotics is a major public health problem and an example of rapid adaptation under natural selection by antibiotics. The dynamics of antibiotic resistance within and between hosts can be understood in the light of mathematical models that describe the epidemiology and evolution of the bacterial population. “Between‐host” models describe the spread of resistance in the host community, and in more specific settings such as hospitalized hosts (treated by antibiotics at a high rate), or farm animals. These models make predictions on the best strategies to limit the spread of resistance, such as reducing transmission or adapting the prescription of several antibiotics. Models can be fitted to epidemiological data in the context of intensive care units or hospitals to predict the impact of interventions on resistance. It has proven harder to explain the dynamics of resistance in the community at large, in particular because models often do not reproduce the observed coexistence of drug‐sensitive and drug‐resistant strains. “Within‐host” models describe the evolution of resistance within the treated host. They show that the risk of resistance emergence is maximal at an intermediate antibiotic dose, and some models successfully explain experimental data. New models that include the complex host population structure, the interaction between resistance‐determining loci and other loci, or integrating the within‐ and between‐host levels will allow better interpretation of epidemiological and genomic data from common pathogens and better prediction of the evolution of resistance.

## 1 INTRODUCTION

The evolution of resistance to antibiotics in bacterial pathogens is an example of rapid evolution in action. Since the introduction of antibiotics in the 1940s, resistances to multiple antibiotics have emerged and spread in bacterial species. Antibiotic resistance in bacteria is an important public health problem (*Antimicrobial resistance global report on surveillance: 2014 summary*, 2014), as infection with resistant strains leads to prolonged hospital stay and increased risk of death (Chang et al., 2011; Cosgrove et al., 2003; de Kraker, Davey, & Grundmann, 2011; De Kraker et al., 2010; DiazGranados, Zimmer, Mitchel, & Jernigan, 2005).

### 1.1 The evolution of antibiotic resistance, an interesting challenge for evolutionary biology

Understanding the evolution of antibiotic resistance is an interesting challenge for evolutionary biology for two main reasons. First, as resistance is an important public health concern, a lot of data on the evolution of resistance is collected. These data can be used to precisely inform and test models. For example, surveillance programmes provide data on the frequency of different types of resistance in various bacterial species infecting humans, in many countries and over several decades. Genomic data sets reveal how and when genetic determinants of resistance evolve by mutation and horizontal gene transfer (Baker, Thomson, Weill, & Holt, 2018). Experiments in vitro or in animal models can be used to follow the within‐host evolution of resistance over a few days or weeks (Singh & Tam, 2011). Second, models of resistance evolution are conceptually interesting. As already argued elsewhere (Read & Huijben, 2009), the evolution of drug resistance is not a simple process of mutant emergence and fixation. Instead, models of resistance evolution need to consider various heterogeneities in the host population, different levels of selection (within and between hosts), and the interplay between the demography of the pathogen (prevalence of the infection at the population level; demography within the host) and the evolution of resistance. These processes generate complex and interesting dynamics described in this review.

### 1.2 Early models of antibiotic resistance evolution

The concepts of evolutionary biology can be used to understand and predict the emergence and spread of antibiotic resistance in bacteria. These concepts can be formalized into mathematical models. Models generate predictions that can be compared with data and extrapolated to predict, for example, the future evolution of resistance or the impact of public health interventions. Early models of antibiotic resistance evolution have been designed from the 1970s (Krus & Rvachev, 1971; Massad, Lundberg, & Yang, 1993), but more influential models were formulated from the late 1990s (Bonhoeffer, Lipsitch, & Levin, 1997; Levin et al., 1997; Lipsitch & Levin, 1997). Since then, models of antibiotic resistance evolution have flourished, often building on classical differential equation compartmental epidemiological models (Kermack & McKendrick, 1927) popularized by Anderson and May (1991). These models describe the evolution of a bacterial population composed of a sensitive (“S”) and a resistant (“R”) strain colonizing a host population (Box 1). Models have been developed to describe the epidemiology and evolution of antibiotic resistance both at the within‐ and between‐host levels. These models are primarily concerned with “microevolutionary” timescales of a few days to months (within host) to decades (between host).

Box 1 **The structure of evolutionary epidemiology models**

**Figure 1:**
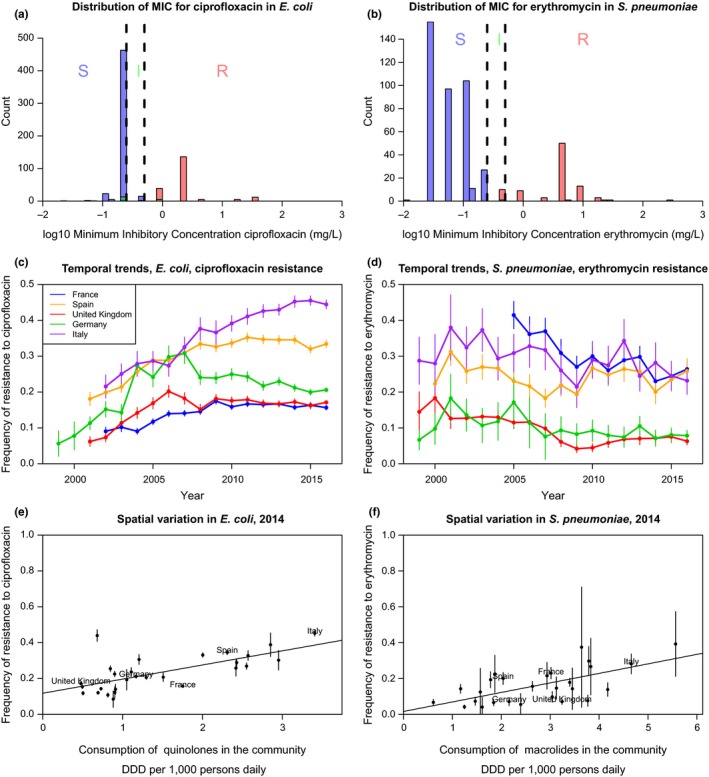
The distribution of minimum inhibitory concentration (MIC), temporal and spatial trends in resistance in Europe over the last 30 years (data from the European Center for Disease Prevention and Control or ECDC), for two example bacterial species and resistances. On the left panels (a, c, e), resistance to ciprofloxacin (a quinolone) in *Escherichia coli*, a Gram‐negative commensal colonizing the gut of virtually all humans, as well as domestic and wild animals and persisting in the environment, and an opportunistic pathogen causing infections responsible for about a million death each year (Denamur, Picard, & Tenaillon, 2010). On the right panels (b, d, f), resistance to erythromycin (a macrolide) in *Streptococcus pneumoniae*, a Gram‐positive colonizing the nasopharynx of children and the elderly, specialized on humans, causing infections responsible for about a million death each year (O'Brien et al., 2009). The top panels (a, b) show the distribution of the minimum inhibitory concentration at year 2016. The colour of the bar denotes the resistance status (I: intermediate; R: resistant; S: sensitive) according to the EUCAST breakpoints (vertical dashed lines). The resistance status may not match exactly because of misclassification and/or conflicting results between different tests. The middle panels (c, d) show the temporal trends in the frequency of resistance in five large European countries (vertical lines show the 95% binomial confidence intervals). The bottom panels (e, f) show the correlation between the frequency of resistance and the consumption of the relevant class of antibiotic in the community across European countries (vertical lines show the 95% binomial confidence intervals)

What trait is being modelled? Models often consider the evolution of two strains, the resistant and the sensitive strain. The resistance status of a strain can be experimentally determined, for example by growing the strain on a gradient of antibiotic concentrations. The lowest concentration above which a bacterium does not multiply to form a colony is called the minimum inhibitory concentration (MIC), and a strain is classified as “Resistant” if its MIC is above a breakpoint. The breakpoints are not standardized: for example a set of breakpoints is defined by the European Society of Clinical Microbiology and Infectious Diseases (EUCAST), another by the Clinical and Laboratory Standards Institute (CLSI). The distribution of MIC across strains in a population is often bimodal, justifying the classification in the two discrete categories sensitive “S” and resistant “R” (Figure 1) in most modelling studies (but see Opatowski et al., 2010; Temime, Boëlle, Courvalin, & Guillemot, 2003 modelling the evolution of the whole MIC distribution).

**Figure 2:**
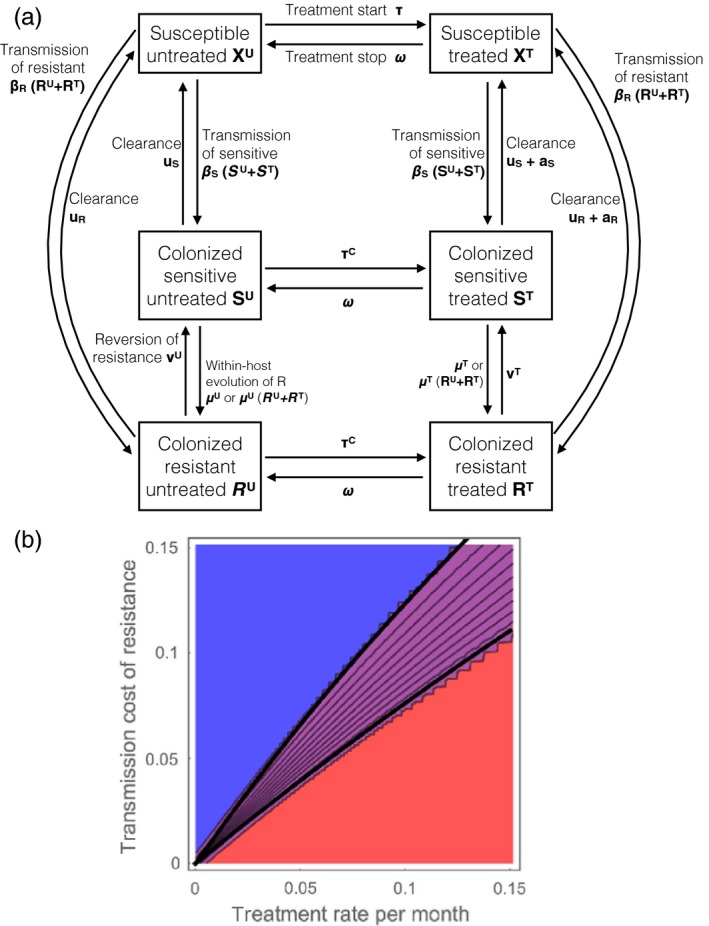
The structure of epidemiological models of antibiotic resistance evolution. (a) The flow diagram of the model. The model follows the dynamics of six variables corresponding to the densities of individuals uncolonized, colonized by S and colonized by R, and untreated or treated. (b) The equilibrium frequency of resistance in model “a” (0 in blue, intermediate in purple and 1 in red), as a function of the cost of resistance on transmission and the treatment rate per month. Black lines are analytical coexistence conditions found by invasion analysis. Coexistence is defined as the frequency of resistance and sensitive strains being above 0.01. In this model, coexistence is maintained in a narrow range of parameters unlike what is observed in data (Figure 1). Within‐host evolution and reversion of resistance are considered rare enough to be negligible (*μ* ^U^ = *μ* ^T^ = 0, *ν* ^U^ = *ν* ^T^ = 0), but increasing these rates would enlarge the coexistence region. Other parameters, shown on panel (a), are: *β*_S_ = 10, *β*_R_ = 10(1 − *c*), where *c* varies along the *y*‐axis in panel (b), *u*_S_ = *u*_R_ = 1, *τ* = *τ^C^* varies along the *x*‐axis, *ω* = 4, *a*_S_ = 10, *a*_R_ = 0.1. All these parameters are rates expressed in units of month^‐1^

The epidemiology of the bacteria is typically described using classical compartmental models (Anderson & May, 1991; Kermack & McKendrick, 1927). Specifically, the host population is compartmented in susceptible and colonized hosts and the densities of different types of hosts are modified by the events of transmission and natural clearance of bacteria. Hosts colonized by bacteria are not necessarily *infected*, as many species of interest live most of the time a commensal lifestyle and are asymptomatic. Models assume there is no specific immunity preventing further colonization, so there is no “recovered” compartment. A system of ordinary differential equations describes the dynamics of the densities of each type of host. To specifically study the evolution of antibiotic resistance, the hosts are subdivided into hosts colonized by a resistant strain and those colonized by a sensitive strain, and all hosts may be subdivided into untreated hosts and hosts treated with antibiotics (Figure 2a). Some studies consider stochastic versions of this type of model, particularly those interested in the small populations in intensive care units or hospital wards (see paragraph 1.2).

Two pioneering studies defined the major questions in mathematical models of antibiotic resistance evolution: what are the factors favouring the sensitive and resistant strains? What are the conditions for their coexistence? What is the equilibrium frequency of resistance as a function of the treatment rate in the population? The first study (Levin et al., 1997) considered the evolution of a bacterial species that colonizes human hosts and multiplies in the environment, with a low rate of exchange between these compartments. The host‐environment structure maintains both the sensitive and resistant strain. Upon treatment, bacteria within a host instantaneously become fully resistant because of rapid within‐host evolution or replacement of the majority sensitive strain with a minority resistant strain. After treatment, bacteria evolve back to full sensitivity because resistance is costly. Similarly, the frequency of resistance in the environment constantly declines. In this model, the evolution and reversion of resistance are both extremely rapid, and the epidemiological dynamics are not explicitly modelled, which may explain why the structure of this model was rarely adopted in subsequent studies. In contrast, the second study (Austin, Kakehashi, & Anderson, 1997) more explicitly formalized the epidemiological dynamics within a compartmental model and defined the basic structure that most models of resistance evolution subsequently adopted. It describes the dynamics of untreated and treated hosts colonized by sensitive and resistant commensal bacteria. Antibiotic treatment is assumed to be independent of colonization by the focal species. Thus, the focal species experiences “bystander selection” by antibiotics that are prescribed to treat other viral or bacterial infections, a scenario relevant to many commensal bacterial species (Tedijanto, Olesen, Grad, & Lipsitch, 2018). In this model, coexistence between the two strains is possible in a narrow window of treatment rates, because the treated hosts form a niche in which the resistant strain can multiply. When the treatment rate exceeds the upper value of this narrow window, resistance fixes in the population. Later on, this model was fitted to data to quantify the impact of a change in antibiotic consumption on the dynamics of resistance (Austin, Kristinsson, & Anderson, 1999).

These two models, and in fact the vast majority of models of resistance evolution, include a *cost of resistance*. If resistance only conferred a benefit under antibiotic treatment, resistance variants would eventually sweep to fixation. Fixation of resistant strains is prevented in models by assuming that resistant strains transmit less, are cleared faster by the host or are less able to super‐colonize an already colonized host. The cost of resistance mutations on the growth rate of bacteria can be measured in vitro. A cost was often found, but not always (Melnyk, Wong, & Kassen, 2015), perhaps because it sometimes acts on components of fitness that are not revealed by these assays.

### 1.3 Aim of the review

Models similar to Austin et al.’s have rapidly been developed to study a diversity of questions at different scales, including the spread of resistance within and between hospitals (Austin & Anderson, 1999), best treatment strategies to limit the spread of resistance (Bonhoeffer et al., 1997) and the evolution of resistance within the host (Austin & Anderson, 1999; Lipsitch & Levin, 1997). This family of models is the main focus of this review (Box 1). The first part reviews models of the transmission and evolution of resistance at the epidemiological level (between hosts). The second part reviews the models of resistance evolution within the host. I particularly focus on the conceptual contributions of these models, and how they allow the interpretation of epidemiological, genomic and experimental data, the design of strategies to limit the spread of resistance, and the prediction of the dynamics of resistance.

## 2 BETWEEN‐HOST MODELS OF ANTIBIOTIC RESISTANCE EVOLUTION

### 2.1 Models of resistance evolution in the community

Many models have been developed to describe the transmission of sensitive and resistant bacterial strains between hosts (reviewed in Opatowski, Guillemot, Boëlle, & Temime, 2011; Spicknall, Foxman, Marrs, & Eisenberg, 2013; Temime, Hejblum, Setbon, & Valleron, 2008). In these models, the competition between sensitive and resistant strains (“S” and “R” strains) is a crucial determinant of the dynamics of sensitive and resistant strains. Indeed, the sensitive and resistant strains compete to colonize the same available hosts, usually resulting in fixation of the fittest. This is true not only in simple models but also in models with additional complexities (Colijn et al., 2010).

The prediction of competitive exclusion of one of the strains differs from the patterns observed in surveillance data: most types of resistance in most species are stable at an intermediate frequency over decades, or on the way to stabilization (Figure 1). These empirical patterns cannot be explained by a very slow selective sweep of a resistant strain over several decades, as the frequency of resistance evolves very fast under changes in selective pressures caused by seasonal changes in the rates of antibiotic prescription (Blanquart, Lehtinen, & Fraser, 2017; Dagan et al., 2008). One important challenge is to formulate models that reproduce this stable coexistence. Several processes have been hypothesized to stabilize the coexistence of sensitive and resistant strains: below I review five of these processes and evaluate their biological plausibility.

First, rapid within‐host evolution of resistance in treated hosts, and reversion to sensitivity in untreated hosts, would allow coexistence in theory because both S and R strains would constantly be generated by these processes. Genomic data reveal that for many bacterial species, these processes are negligible over epidemiological timescales. Resistant strains form clones that are stable over years to decades (Box 2). This observation is not compatible with the idea that resistance often evolves de novo in treated hosts and reverts in untreated hosts.

Box 2 **Insights from genetic and genomic data**

Genomic data inform models of antibiotic resistance evolution. Bacterial genomes are composed of the core genome (genes shared by all strains of a species) and the accessory genome (genes that are present or absent). Variants conferring antibiotic resistance can either be mutations on genes in the core genome (as for fluoroquinolone resistance mutations for example) or resistance genes that are present or absent. Resistance variants may circulate between different strains by horizontal gene transfer mediated by the mechanisms of transformation or conjugation (if the resistance gene is on a plasmid). Resistance variants are very well‐characterized in species such as *Escherichia coli*, to the extent that resistance can be predicted from whole‐genome sequences (Stoesser et al., 2013). The comparison of genome sequences informs on the rate at which resistance genes are exchanged between strains, on the evolution of resistance within hosts, and on routes of transmission of resistant strains.

**Are resistance gene transfers important at microevolutionary timescales?**

Some models include gains and losses of resistance gene resulting in the conversion of a sensitive into a resistant strain and vice‐versa. Genomic data from several species suggest that gains and losses occur at low rates compared to epidemiological rates and are negligible on microevolutionary (or epidemiological) timescales of a few decades. For example, in *E. coli*, a clone (called ST131 group C2) with a CTX‐M beta‐lactamase resistance gene and a fluoroquinolone resistance mutation emerged in the late 1980s and up to now has maintained a stable assemblage of these resistance genes (Ben Zakour et al., 2016; Kallonen et al., 2017). In *Streptococcus pneumoniae* (Croucher et al., 2013) and *Staphylococcus aureus* (Ledda et al., 2017), resistance is similarly spread by clonal expansion of a handful of successful clones with little gain or loss of resistance. For example, in Ledda et al. (2017), among 17 *S. aureus* isolates evolving from a common ancestor for around 35 years, resistance was gained twice. Among 213 isolates spanning 24 years of evolution, resistance was lost seven times. Thus, large genomic data sets collected in common species suggest that successful horizontal gene transfers are rare at timescales of a few years to a few decades. Intriguingly, this is in contrast with experimental studies in humans and animals showing that horizontal gene transfer can happen over a few days (reviewed in Hoelzer et al., 2017). Possibly, horizontal gene transfer occurs frequently within hosts but few new strains created by such transfers are fit and successfully transmit onward. Lastly, gene transfer between species is also rare: the CTX‐M gene is originally a chromosomal gene of the commensal species *Kluyvera*, and nine introduction events in *E. coli* have been detected since the 1980 s or earlier (Cantón, González‐Alba, & Galán, 2012).

**How frequent is de novo evolution of resistance by point mutations within treated hosts?**

Genetic data allow tracking the evolution within the host and identifying whether the resistance in a host is *primary* or transmitted (i.e., imported by existing resistant strains colonizing the host) or *acquired*, that is evolving de novo by point mutation within the host. The evolution of resistance de novo over a typical antibiotic course seems rare. For many common species, the within‐host evolution of resistance was only observed in clinically exceptional infections of long duration often resulting in the patient's death, for example, in infections caused by *E. coli* (Dupont et al., 2017; Rasheed et al., 1997), *S. aureus* (Mwangi et al., 2007) and *Salmonella enterica* Typhimurium (Blair et al., 2015). This may be due to a detection bias, as it is hard to establish longitudinal follow‐up and sampling of individuals before and after a short antibiotic course of 1–2 weeks. But it may also point to the genuine rarity of de novo evolution of resistance over a short time period. The latter explanation is supported by a recent study of 51 patients with *S. aureus* bacteraemia, treated for a duration of 26 days on average, where an increase in vancomycin resistance was only found in five episodes in blood isolates (Giulieri et al., 2018). In other species causing long infections, resistance can be acquired via chromosomal mutation and the within‐host evolution of resistance is well documented, with interesting phenomena of competition between multiple selected mutations (clonal interference) within the host, as well as local differentiation of the bacterial population. This is the case in chronic lung infections caused by the species *Mycobacterium tuberculosis* (Eldholm et al., 2014; Gygli, Borrell, Trauner, & Gagneux, 2017), and *Pseudomonas aeruginosa* in cystic fibrosis (Winstanley, O'Brien, & Brockhurst, 2016). Despite frequent de novo evolution in *M. tuberculosis*, some resistant clones are transmitted onward and circulate in the population, resulting in >90% of resistance being primary (Kendall, Fofana, & Dowdy, 2015; Luciani, Sisson, Jiang, Francis, & Tanaka, 2009).

**Is resistance in animal hosts and the environment important for resistance in humans?**

The impact of resistant bacterial strains circulating in farm animals or the environment on resistance in humans depends on the rate of transmission between these reservoirs, which can in principle be inferred from genomic data. Studies based on genetic markers revealed a broad genetic similarity between chicken and human *E. coli* strains (Johnson et al., 2006, 2007; Kluytmans et al., 2013; Leverstein‐van Hall et al., 2011; Overdevest et al., 2011), suggesting frequent transmission. However, this apparent genetic similarity was explained by the poor resolution of genetic techniques, and analysis of whole genomes actually revealed considerable genetic differences. Strains collected in chicken and humans and previously supposed to be identical were actually separated by 1,263 single nucleotide polymorphisms (SNPs) (de Been et al., 2014). In contrast, in the same study, pig and human strains from a single zoonotic disease outbreak were separated by only 0–6 SNPs. That study also revealed that the *plasmids* circulating in chicken and humans were extremely similar (separated by 0–4 SNPs). Greater similarity in plasmids does not imply that inter‐species plasmid transfer is particularly more frequent than bacterial transmission because the difference can be simply explained by the much smaller size of plasmids (50 kb) compared to the whole genome (5 Mb). Another study found chicken and human strains as close as 70 SNPs apart (Falgenhauer et al., 2016). It is difficult to conclude on the rates of transmission within versus between species from these studies, because the number of SNPs separating closest strain also depends on the density of the sampling (denser sampling allows finding closer strains) and potential sampling biases (Singer, 2015). In the future, an interesting perspective would be to adapt phylogeographic methods (De Maio, Wu, O'Reilly, & Wilson, 2015) to robustly infer epidemiological parameters (rates of transmission) from phylogenetic trees of bacteria sampled in different hosts (Muloi et al., 2018). This will be allow a better interpretation of the new larger data sets (Ludden et al., 2018).

**Phylodynamics of resistance evolution**

Phylodynamics is the discipline that reconstructs the epidemiological and evolutionary dynamics of a population based on its phylogenetic history (Grenfell et al., 2004). Applied to bacterial populations, these techniques allow reconstructing the history of mutations or gene acquisitions, as was done for example for the resistant *E. coli* ST131 clone (Ben Zakour et al., 2016; Price et al., 2013) or *S. pneumoniae* PMEN2 clone (Croucher et al., 2014). The history of geographic spread of these lineages may also be inferred, albeit with little power when global spread was rapid (Petty et al., 2014; Price et al., 2013). The history of population size and growth rates of particular lineages can also be inferred using the coalescent framework, but recombination and selection may bias these techniques (reviewed in Lapierre, Blin, Lambert, Achaz, & Rocha, 2016). Lastly, an interesting perspective would be to develop new phylodynamics techniques to infer selection on resistant and sensitive strains.

Second, coinfection of a host by both the S and R strains stabilizes coexistence. However, models with coinfection often implicitly assume ecological differentiation between the resistant and sensitive strain within hosts (Lipsitch, Colijn, Cohen, Hanage, & Fraser, 2009). Models often consider hosts colonized by the S, the R, and both S and R strains, but ignore potential “SS” and “RR” coinfection. Thus, implicitly, there are two distinct ecological niches for the S and R strains within hosts because it is possible for a R strain to invade a host colonized by S, but it is not possible for a R strain to invade a host colonized by R (and reciprocally). The existence of these two niches does not seem biologically plausible for two strains that differ only in their resistance to antibiotics.

Third, hosts treated with antibiotics form a niche in which the resistant strain preferentially replicates. When the sensitive strain is at equilibrium in the host population, a rare resistant strain increases in frequency by colonizing treated hosts, then stabilizes at an intermediate frequency. However, this niche is small and transient because antibiotics are typically prescribed for a few days to weeks, and this mechanism explains little coexistence (Austin et al., 1997; Blanquart, Lehtinen, Lipsitch, & Fraser, 2018). The transient nature of this niche is reflected in the high rate of treatment cessation *ω* on Figure 2a. The narrow range of coexistence explained by this mechanism is depicted on Figure 2b.

Fourth, the stratification of the host population in several classes taking antibiotics at different rates promotes the maintenance of both strains. For example, if the rates of antibiotic prescription are very different across countries and transmission between countries is much smaller than within countries, both R and S strains will be maintained, and the R strain will be at a high frequency in countries with a high prescription rate and at a low frequency in countries with a low prescription rate (Figure 1e,f). This mechanism is powerful when the host classes are very isolated one from another (Blanquart et al., 2018).

Lastly, “genetic differentiation” between S and R strains promotes coexistence: resistance genes may be associated with loci that are themselves under balancing selection and stably coexisting in the population, and that interact with resistance. This hypothesis was verified in *S. pneumoniae*, where different capsular types (serotypes) are stably maintained by the interaction with serotype‐specific host immunity and determine the duration of carriage (asymptomatic colonization). Carriage duration is theoretically predicted to be positively associated with resistance, a prediction verified in several data sets (Lehtinen, Blanquart, Croucher, et al., 2017). More generally, ecologically important bacterial traits with stable genetic diversity (Levin, 1988) could be interacting with resistance, which would promote the evolution of resistant and sensitive clones stably coexisting and associated with some of these traits.

In summary, current theory suggests the coexistence of S and R strains may be explained not by a single mechanism but by a combination of several processes: the niche formed by treated hosts, the host population structure, and the association with loci themselves under negative frequency‐dependent (also called “balancing”) selection (Cobey et al., 2017).

Perhaps because of the difficulty of formulating simple and plausible dynamical models reproducing the stable coexistence of sensitive and resistant strains, almost no studies fit dynamical models to data on the frequency of resistance in the community. Austin, Bonten, Weinstein, Slaughter, and Anderson (1999) and Austin, Kristinsson, et al. (1999) fitted a model to data on resistance in several countries, but the model had an implausible fitness trade‐off whereby the resistant strain transmits more, but may be super‐colonized by the sensitive strain (Lipsitch et al., 2009). A notable exception is the study of the dynamics of resistance in *Neisseria gonorrhoeae*, an important sexually transmitted pathogen mostly affecting men having sex with men (MSM), in the United Kingdom from the 1990s to 2015. Fingerhuth et al. fitted a compartmental model to data on the frequency of ciprofloxacin and cefixime resistances from 1995 to 2010, showed that both resistances spread faster in MSM than in heterosexual mean and inferred that MSM experience a higher rate of antibiotic treatment (Fingerhuth, Bonhoeffer, Low, & Althaus, 2016). During that first period, both resistances increased approximately exponentially and most diagnosed cases were treated with cefixime. The use of cefixime peaked in 2008 and then declined because of increased resistance and treatment failure leading to a change of the first line treatment. Resistance to cefixime in MSM peaked in 2010 and subsequently declined to 0. Whittles et al. fitted a compartmental model to data on that second phase of evolution (2005–2015) and explained the decline in cefixime resistance when cefixime use was reduced by a cost of resistance. More precisely, the recovery rate from cefixime‐resistant infection was inferred to be twice as fast as that from sensitive infections (Whittles, White, & Didelot, 2017). In that context, there was no long‐term coexistence between cefixime‐resistant and cefixime‐sensitive strains, as cefixime resistance transiently emerged, peaked and went extinct.

Most models of antibiotic resistance evolution I described so far make the assumption that the host population is homogeneous. However, bacteria do not evolve in a homogeneous host population: some species may colonize human hosts, wild animals or farm animals, and persist or multiply in the environment (surfaces, water, soil, air, etc.). Human hosts themselves are varied, as bacteria may colonize healthy individuals in the community or individuals in hospitals. A particular attention has been paid to the evolution of resistance in hospitals, and in farm animals, two environments where antibiotic use is frequent.

### 2.2 Selection for resistance in hospital settings

A large number of models have focused on the evolution of antibiotic resistance specifically in hospitals, or intensive care units or wards within hospitals. Hospitals have several distinct properties compared to the community: the rate of antibiotic treatment is high, the host population size is small (in the order of 10s of people), and there is a very rapid turnover caused by the admission and discharge of patients (the mean length of stay in OECD countries is 8 days Health at a Glance 2017, 2017). Most models are specifically concerned with methicillin‐resistant *S. aureus* (MRSA) and vancomycin‐resistant enterococcus, two Gram‐positive antibiotic‐resistant pathogens (van Kleef, Robotham, Jit, Deeny, & Edmunds, 2013) and thought to be particularly selected for in hospitals. Fewer models describe extended spectrum beta‐lactamase Gram‐negative bacteria, which are common both in the community and in hospitals. Models have been formulated at various levels, from single ward or units (Cooper, Medley, & Scott, 1999; Sébille, Chevret, & Valleron, 1997) to hospitals (Lipsitch, Bergstrom, & Levin, 2000) and networks of hospitals linked with the community (Smith, Dushoff, Perencevich, Harris, & Levin, 2004).

Two assumptions of hospital models seem critical to the evolutionary outcome. Many models only consider hosts who are uncolonized, or colonized by a resistant strain, but not hosts colonized by a sensitive strain (Austin, Bonten, et al., 1999; Austin, Kristinsson, et al., 1999; Cooper et al., 1999, 2004; D'Agata, Webb, & Horn, 2005; Pelat et al., 2016; Pelupessy, Bonten, & Diekmann, 2002; Smith et al., 2004). These models are, in fact, population dynamics models without evolution, and the competition between the sensitive and the resistant strain is not modelled. The second crucial assumption of hospital models is the frequency of resistance in incoming patients and the intensity of selection for resistance in the hospital. It is commonly assumed that incoming patients are mainly colonized by the sensitive strain and resistance is strongly selected in the hospital (Lipsitch et al., 2000). However, in other models, incoming patients carry the resistant strain and substantially contribute to the persistence of resistance in the hospital (Austin, Bonten, et al., 1999; Austin, Kristinsson, et al., 1999; D'Agata et al., 2005).

**Figure 3:**
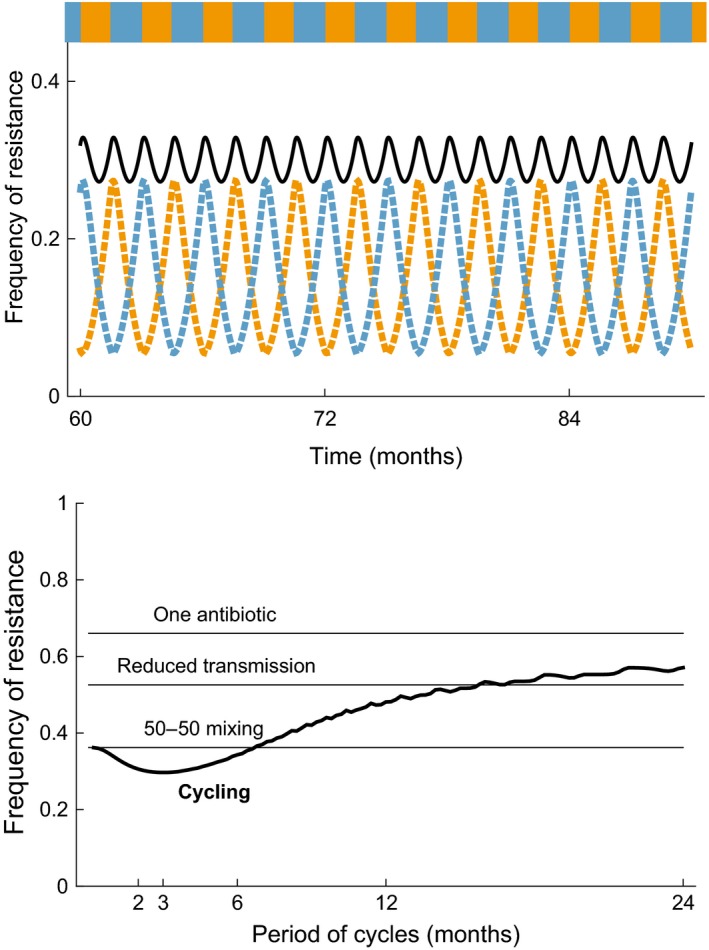
The equilibrium frequency of resistance in a hospital, as a function of the antibiotic prescription strategy. The model is analogous to that in Figure 2, extended to two resistances and two corresponding treatments (Appendix S1). The top panel shows the frequency of resistance to the first (orange dashed line) and second (light blue dashed line) antibiotic, and the total frequency of resistance (black line) as a function of time, for a cycling strategy of period 3 months. The bottom panel shows the time‐averaged frequency of resistance under the cycling strategy (thick line) as a function of the cycling period. For comparison, horizontal lines show the frequency of resistance when only one antibiotic is constantly prescribed, when one antibiotic is prescribed with transmission in the hospital reduced by 50% (demonstrating the effect of reducing transmission on the frequency of resistance mentioned in paragraph of Section 2.1), and under the 50%‐50% mixing strategy. The incoming patient population is assumed uncolonized or colonized by the sensitive strain, and the total rate of antibiotic treatment in the hospital is high, at 3 month^−1^. Other parameters are as follows: rate of admission and discharge 1 month^−1^, and total hospital population assumed constant. Transmission and clearance parameters as for Figure 2 for both strains

Most hospital models examine the impact of intervention measures to reduce resistance (reviewed in van Kleef et al., 2013). When an influx of resistant strains is necessary for the persistence of the resistant strain in the hospital, reducing the influx of colonized patients reduces resistance (D'Agata et al., 2005). All models show that reducing transmission efficiently reduces resistance. A reduction in transmission can be achieved by hand washing (Cooper et al., 1999; Pelat et al., 2016; Sébille et al., 1997), isolation measures (Cooper et al., 2004) or cohorting of healthcare workers and patients (at the extreme, assigning a single healthcare worker to each patient) (Austin, Bonten, et al., 1999; Austin, Kristinsson, et al., 1999). It might appear obvious that reducing transmission reduces resistance. It is, indeed, when only the resistant strain (and not the sensitive) is modelled, as in a pure population dynamics model a reduction in transmission will result in a reduction in prevalence of the resistant strain. When both the sensitive and resistant strains are considered, it is less intuitive why a reduction in transmission would harm the resistant more than the sensitive strain. This is because the resistant strain multiplies mainly by transmission in the hospital, while the sensitive strain is sustained by immigration from the community (Lipsitch et al., 2000) (Figure 3).

Other interesting theoretical predictions have been made in the scenario where the resistant strain is strongly selected in the hospital, but most incoming patients are colonized with a sensitive strain (Lipsitch et al., 2000). This is analogous to a model of adaptation to a “sink” habitat in “source‐sink” ecological models. The source—the favourable habitat—is the community dominated by the sensitive strain. The source continuously sends individuals to the sink. The sink—the unfavourable habitat—is the hospital, where the sensitive strain is unfit but the resistant strain spreads and allows persistence of bacteria. Because of the fast turnover and the influx of sensitive strains, any intervention to reduce resistance in the hospital has a very fast effect (~ weeks). Indeed, if resistance is no longer favoured in the hospital, resistant strains will rapidly be washed away by the influx of hosts colonized by sensitive strain. The source–sink dynamics have counterintuitive effects on the impact of an intervention at the individual level. If a new antibiotic to which both the sensitive and resistant strains are sensitive is used in the hospital, both strains will be cleared by treatment, reducing the prevalence of resistance at the population level. However, at the individual level, hosts who have been treated with the new drug are more likely to be carrying a resistant strain because they may be recolonized by the resistant strain endemic in the hospital. Thus, this intervention, beneficial at the population level, may appear to favour resistance at the individual level.

Hospitals are linked with other hospitals and embedded in the community. Networks of hospitals create metapopulation dynamics (Smith et al., 2004) where the prevalence of resistance is determined jointly by migration of patients between hospitals and transmission within hospitals. More generally, as resistance emerges, it will spread faster in “core groups” where resistance is favoured, such as hospitals, long‐term care facilities or older age classes (Smith et al., 2004). Moreover, when the competition between sensitive and resistant strains is considered, interactions between the community, hospitals and different risk groups allow the coexistence of multiple strains (Kouyos, Klein, & Grenfell, 2013).

At the level of small units such as hospital wards or intensive care units, stochastic effects are important. Several realizations of the same stochastic model exhibit considerable variability (Cooper et al., 1999). Resistance levels fluctuate stochastically, and resistant strains sometimes go extinct, which weakens the response to antibiotic selection and may explain the observation that smaller hospitals have a lower frequency of methicillin‐resistant *S. aureus* (Kouyos, zur Wiesch, & Bonhoeffer, 2011a).

Lastly, in the context of small extensively monitored units, detailed parameterization and model fitting is possible (Austin, Bonten, et al., 1999; Austin, Kristinsson, et al., 1999; Grundmann, Hori, Winter, Tami, & Austin, 2002). According to a meta‐analysis, about 35% of modelling studies (including both deterministic and stochastic models) fitted the model on data and 5% have validated the model on a distinct data set (van Kleef et al., 2013). These studies thus inferred important epidemiological parameters such as transmission, evaluated the importance of different routes of transmission or predicted the impact of interventions.

### 2.3 Evolution of resistance in animal hosts and the human community

There has been comparatively less modelling on the impact of antibiotic consumption by farm animals on the evolution of antibiotic resistance in humans. An influential model (Smith, Harris, Johnson, Silbergeld, & Morris, 2002) showed that the impact of agricultural antibiotic use on the frequency of resistance in humans crucially depends on the ability of the bacteria to spread in humans. In this model, agricultural antibiotic use is implicitly modelled by a constant influx of the resistant strain from animals and the environment to humans, representing for example transfer of resistant bacteria via food ingestion. This model only considers resistant strains and lacks the competition with sensitive strains, but a recently developed model with competition complements these findings (Blanquart et al., 2018). Three scenarios are possible:

1. If the resistant strain cannot spread epidemically in the human population on its own, the dynamics of the system is analogous to a “source–sink” dynamics, and the frequency of resistance in humans (the sink) will be strongly affected by the frequency of resistance in animals (the source). This corresponds to zoonotic pathogens such as *Campylobacter* or *Salmonella* (Lipsitch, Singer, & Levin, 2002).
2. If resistance has not yet fully emerged and spreads slowly in humans, while it is at a higher frequency in farm animals, the influx of resistant strains from animals can considerably accelerate the emergence of resistance in humans. In that case, farm animals are “incubators” of resistance. For example, the mcr‐1 gene conferring colistin resistance to *E. coli* was first discovered in chicken and pigs in China (Liu et al., 2016) and also identified in human *E. coli* strains (Hu, Liu, Lin, Gao, & Zhu, 2016). The higher frequency of this gene in *E. coli* from farm animals and the more frequent use of colistin in agriculture suggest that this resistance initially emerged and spread in animals.
3. If the resistant strain can spread and persist in humans, it will reach an equilibrium frequency. In that scenario, if the transmission of bacteria from animals to humans occurs at a *very* low rate, the equilibrium frequency in humans will be almost unaffected by that in animals. However, low rates of transmission between animal and human hosts are sufficient to homogenize the populations to an intermediate value of resistance. These predictions result from a general model of evolution in a structured host population (Blanquart et al., 2018) where the host classes represent human and animal hosts.

In practice, the scenario (iii) is relevant to many commensal bacterial species (e.g., *E. coli*) where the bacteria can persist in humans and resistance has already emerged. In these species, is the transmission rate between animals and humans high enough for resistance in farm animals to impact levels of resistance in humans? Transmission of bacteria between farm animals and farm workers is extensively documented for common bacterial species (Hoelzer et al., 2017; Tang et al., 2017). But both genomic (Box 2) and epidemiological data suggest that transmission rates from farm animals to the wider community are low. Antibiotic use in farm animals was suspected to contribute to the rise of vancomycin‐resistant enterococci (VRE) in humans (Smith, Dushoff, & Morris, 2005), but the epidemiological evidence is scarce. It is well established that the use of avoparcin, an antibiotic used as a growth promoter in farm animals in Europe, selected for vancomycin resistance in animals. Resistance in animals generally declined in several countries following bans on the use of antibiotics as growth promoters in the 1990s (Aarestrup et al., 2001; Boerlin, Wissing, Aarestrup, Frey, & Nicolet, 2001; Emborg et al., 2003; Van den Bogaard, Bruinsma, & Stobberingh, 2000). A decline in VRE has been concomitantly documented in healthy humans carriers in only two studies (Klare et al., 1999; Van den Bogaard et al., 2000). More broadly, resistance in *E. coli* from sampled in farm animals was shown to be correlated with resistance in *E. coli* sampled in humans across European countries (Vieira et al., 2011), but this pattern may be explained by correlations in antibiotic use in farm animals and humans across countries.

### 2.4 Best antibiotic prescription strategies: cycling, mixing and combination therapy

An interesting debate arose regarding the optimal antibiotic prescription strategy to manage infections in small well‐defined populations subjected to high rates of treatment, such as intensive care units or hospital wards. The optimal strategy is usually defined as the strategy minimizing the total number of colonized patients over a defined time period. The debate focused on strategies based on the deployment of two drugs in the presence of two types of resistances, and particularly on the relative merits of two strategies: (a) the “mixing” strategy, whereby at each time point a fraction of individuals receives antibiotic A while the rest receives antibiotic B, and (b) the “cycling” strategy whereby all individuals at a time point are treated with one antibiotic, which alternates periodically between A and B.

The first two models addressing this question found that mixing outperforms cycling, in the community (Bonhoeffer et al., 1997) and in the hospital (Bergstrom, Lo, & Lipsitch, 2004). The same intuition underlies the result in both models. Consider the simple scenario where the two resistant strains are equivalent in their costs and the two antibiotics are prescribed at equal rates. When cycling is slow, bacteria resistant to the current antibiotic increase in frequency and therefore most antibiotic prescription becomes ineffective; in contrast, under 50–50 mixing, half of the antibiotic prescriptions are effective. Actually, both the cycling and the mixing strategies were outperformed by combination therapy, whereby both antibiotics are prescribed to everyone, provided the rate of de novo evolution of dual resistance is not too high (Bonhoeffer et al., 1997). This last finding was later on confirmed within a more general model (Tepekule, Uecker, Derungs, Frenoy, & Bonhoeffer, 2017).

More recently, and in constrast to these early findings, optimal control theory revealed that for a general class of ordinary differential equation models, there is always a cycling strategy that outperforms the best mixing strategy (Peña‐Miller & Beardmore, 2010a). Indeed, extremely rapid cycling is equivalent to mixing, so the best cycling must be at least as good as mixing. The applicability of these results in clinical practice has been questioned, however, because the optimal cycling strategy is hard to find and requires perfect knowledge of the dynamical system. A sub‐optimal but realistic “responsive cycling” strategy—regularly probing the state of the system and switching to the drug with least prevalent resistance—outperforms the cycling strategy if probing is frequent enough (Bonhoeffer, zur Wiesch, Kouyos, 2010; Peña‐Miller & Beardmore, 2010b).

How useful are these theoretical results for clinical practice? There is no clear empirical evidence on the relative merits of mixing and cycling. In a systematic review of studies on antibiotic cycling efficacy (Brown & Nathwani, 2005), only one study compared the efficacy of cycling to standard practice and revealed no significant difference in the colonization by resistant strains, the incidence of nosocomial infections or mortality (Toltzis et al., 2002). Cycling was better than standard practice in terms of reducing resistance and mortality in hospitals, according to a more recent meta‐analysis of 11 studies (zur Wiesch, Kouyos, Abel, Viechtbauer, & Bonhoeffer, 2014). In a randomized trial conducted in eight intensive care units in five European countries, no difference in the incidence of resistance or the all‐cause mortality was found during cycling versus mixing, suggesting no strong difference between the two strategies (van Duijn et al., 2018), in spite of good adherence to the strategies. This result is somewhat disappointing, but in line with theory predicting that any difference in the outcome of cycling and mixing strategy is small compared to the substantial stochastic variation around the expected outcome (Beardmore, Peña‐Miller, Gori, & Iredell, 2017).

Recent work explored more detailed models and specific responsive strategies. A responsive cycling strategy using measures of resistance to detect if a type of resistance goes extinct, and deploying the corresponding drug, outperforms both mixing and cycling (Kouyos, zur Wiesch, & Bonhoeffer, 2011b). This strategy works only in small units where stochastic effects are important and extinction of a resistant strain is possible. Another “responsive cycling” strategy whereby the drug is switched if the patient becomes symptomatic (which indicates treatment failure in that model) also outperforms both mixing and cycling (zur Wiesch et al., 2014). “Sequential monotherapy,” whereby the drug is adapted at the individual level based on personal resistance tests, outperforms mixing, cycling and responsive cycling (Beardmore et al., 2017).

In spite of more than twenty years of mathematical modelling to assess different treatment strategies, there is scope for better models and theoretical unification. For example, in a model where the treatment status of individuals is explicitly described (as on Figure 2a), “cycling” can be a better strategy than “mixing” (Uecker & Bonhoeffer, 2017; Figure 3). This model explicitly describes untreated and treated hosts, unlike many previous models assuming that the sensitive strain is instantaneously cleared upon treatment and that the resistant strain is unaffected by treatment.

In conclusion, epidemiological (between‐host) models of resistance evolution form a rich and diverse literature but the connection with data is still tenuous, with the exception of models of resistance evolution in the very well‐controlled setting of intensive care units in hospitals. Epidemiological models assume that events happening within the host, such as bacterial clearance by the antibiotic or replacement of a bacterial strain by another, occur on a fast timescale and can be represented by instantaneous transitions between different compartments (Figure 2a). A distinct class of models describes in more details the processes happening within the host.

## 3 WITHIN‐HOST MODELS OF ANTIBIOTIC RESISTANCE

### 3.1 “Hit hard, hit early”: and selection for resistance

Many within‐host models of antibiotic resistance are based on pharmacokinetics/pharmacodynamics (PK/PD), the discipline interested in the dynamics of the drug concentration and its effect within the host, and population genetics. These models very often investigate what type of antibiotic regimen—in terms of dose, duration and timing—is least likely to drive the evolution of resistance. “Hit hard hit early” is a dominant principle introduced by Paul Ehrlich, a pioneer of chemotherapy (Ehrlich, 1913). This principle was not originally designed to prevent the evolution of drug resistance, but rather to rapidly reduce the pathogen population size to improve the efficacy of the drug and to limit pathogenicity. This principle was applied in the context of the evolution of antibiotic resistance and justified by population genetics principles (see zur Wiesch, Kouyos, Engelstädter, Regoes, & Bonhoeffer, 2011).

Two main reasons justify “hitting hard.” A high antibiotic dose ensures that the resistant strains that may be available in the host, either imported by transmission from other hosts or appearing de novo by mutation, are not resistant enough to grow under this high concentration (Baquero & Negri, 1997; Zhao & Drlica, 2001). Conversely, a lower dose increases the probability that an intermediately resistant strain is available, emerges, potentially leading the way for further mutations increasing resistance. A too low concentration can result from prescription of a low dose, imperfect adherence to treatment (Lipsitch & Levin, 1997) or spatial heterogeneity in drug concentration in the body (“refuges” or “sanctuaries”) (Kepler & Perelson, 1998; Lipsitch & Levin, 1998). These ideas are encapsulated in the concept of “mutant selection window” (Baquero & Negri, 1997; Drlica & Zhao, 2007; Zhao & Drlica, 2001). This is the range of antibiotic concentration spanning from the concentration at which the sensitive strain stops growing (the MIC of the sensitive strain) to the concentration where the maximally resistant strain in the host can no longer grow (the “mutant prevention concentration” or MPC) (Olofsson & Cars, 2007; Zhao & Drlica, 2001). Lastly, in addition to reducing the probability that a resistant enough strain is available, a high drug dose will reduce the bacterial population size faster, which will also reduce the probability that a resistant mutant appears in the population (Lipsitch & Levin, 1997).

However, hitting hard is not necessarily the best strategy to limit the *spread* of resistant strains once they have emerged. A high antibiotic dose limits the chance that a resistant enough strain is available, but it also makes selection for resistance stronger. As a result, under a high antibiotic concentration, if a resistant enough strain is present, it will emerge and spread faster in the host. These two phenomena result in a hump‐shaped (or “inverted‐U”) relationship between the rate of emergence of a mutant and the antibiotic dose (Kepler & Perelson, 1998; Lipsitch & Levin, 1997).

“Hitting early” will limit the emergence of resistance when a small fraction of intermediately resistant strains are initially present: these resistant bacteria can be driven to extinction by an early high dose (Lipsitch & Levin, 1997). If the size of the total bacterial population is initially smaller than at the long‐term equilibrium (without treatment), an early antibiotic dose will also limit the bacterial population size (D'Agata, Dupont‐Rouzeyrol, Magal, Olivier, & Ruan, 2008; D'Agata, Magal, Olivier, Ruan, & Webb, 2007), hence limiting pathogenicity and the probability that a resistance mutant appears.

**Figure 4:**
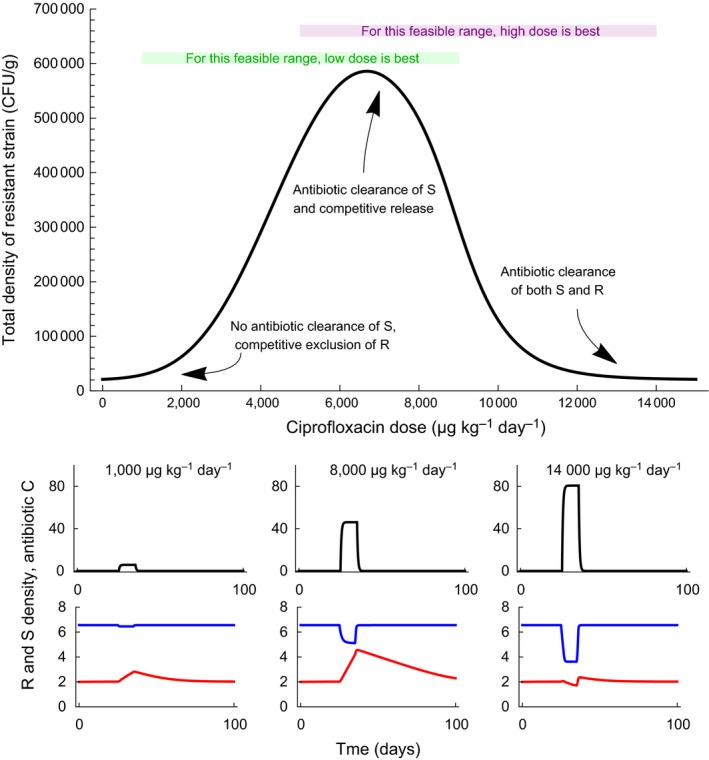
The emergence of resistance is favoured by an intermediate antibiotic dose. This is illustrated with a model describing the evolution of resistance to ciprofloxacin in *Escherichia coli* in piglets under a ciprofloxacin antibiotic course (Nguyen et al., 2014). The top graph shows the total density of resistance (summed over all days), within host, over 100 days including a 10‐days antibiotic course, as a function of the daily antibiotic dose given during these 10 days. The feasible range of doses is limited by practical considerations (clearing the bacteria, toxicity): for the hypothetical range shown in green, the low dose will be best to limit the evolution of resistance; for the purple range, the high dose will be best. The small graphs below give the time course of antibiotic concentration in µg per g of faeces (black line), and of the density of resistant and sensitive strains in log10 colony forming unit (CFU) per g of faeces (red and blue lines) over these 100 days. Under a low antibiotic dose, the sensitive strain is unaffected by treatment and maintains the resistant strain at low density because it has higher competitive ability. At an intermediate dose, the density of the sensitive strain is reduced by antibiotics and the resistant strain is released from competition, then slowly declines after treatment is halted. At a high antibiotic dose, the densities of both the sensitive and the resistant strain are reduced by the antibiotic. The model describes the within‐host antibiotic concentration, and the density of sensitive and resistant strains (Appendix S1). Parameters are as estimated from experimental data in Nguyen et al. (2014), except that here I assume partial sensitivity and resistance of the strains: $C_{S}^{50}=49.1$ μg/g, and $C_{R}^{50}=1,000$ μg/g

Lastly, dose fractionation, the splitting of an antibiotic dose in multiple smaller doses prescribed at more frequent time intervals, will more efficiently kill partially resistant bacteria when the action of antibiotic increases less than linearly with concentration (such as in the model presented in Figure 4) (Lipsitch & Levin, 1997).

### 3.2 Questioning aggressive chemotherapy and emphasizing the role of within‐host competition between sensitive and resistant strains

A series of recent theoretical (Day & Read, 2016; Geli, Laxminarayan, Dunne, & Smith, 2012) and experimental studies of rodent malaria under antimalarial treatment (Huijben et al., 2013; Read, Day, & Huijben, 2011) (reviewed in Kouyos et al., 2014) emphasized the idea that a high drug dose (“hitting hard” or “aggressive chemotherapy”) is not necessarily the best strategy to limit the emergence and spread of resistance within the host. This literature subtly corrects the concept of mutant selection window and emphasizes competition between sensitive and resistant strains within the host. The definition of the mutant selection window is based on *absolute* growth rates of sensitive strains (the lower bound, MIC) and resistant strains (the upper bound, MPC) (Day, Huijben, & Read, 2015). The absolute growth rate of the resistant strain, not its fitness relative to that of the sensitive strain, determines whether the resistant strain will grow within the host (Day et al., 2015). This absolute growth rate is not an intrinsic property of the strain but also depends on the environment in the host, in particular the total bacterial density, the density of immune effectors, etc. At low antibiotic concentrations, the resistant strain cannot grow when resistance is costly, because it is competitively suppressed by the sensitive strain. When the antibiotic concentration gets high enough (at a point which is not the MIC in general), the sensitive strain is removed and this *competitive release* allows the resistant strain to grow (Colijn & Cohen, 2015; Day & Read, 2016) (Figure 4). At concentrations above the MPC, the resistance strain can no longer grow. Although one could imagine a complex and multi‐peaked relationship between the probability of emergence and the antibiotic concentration, in practice it is unimodal in plausible models explored (Day & Read, 2016).

In addition, the antibiotic concentration in the host is limited by practical considerations: it must not be too low, to clear the bacteria efficiently, but it cannot be too high to be practically achieved within the patient without toxic effects (Olofsson & Cars, 2007). Depending on the position of this feasible range with respect to the unimodal relationship, the lowest or highest feasible dose will limit the evolution of resistance (Day & Read, 2016; Kouyos et al., 2014) (Figure 4). If the MPC is lower than the maximal feasible concentration, resistance can be suppressed with aggressive chemotherapy: this might be more common when resistance is acquired by mutations within the host (e.g., fluoroquinolone resistance). On the contrary, if the MPC is much higher than the maximum feasible concentration, moderate chemotherapy and competitive suppression by the sensitive strain may be able to contain the outgrowth of resistance in the host: this situation may be more common when highly resistant strains circulate in the population and resistance is mostly primary.

This theory makes the prediction that aggressive chemotherapy is not always optimal with respect to the emergence of resistant strains. This prediction is verified in in vivo and in vitro systems (see the mini‐review in Day & Read, 2016). A stronger prediction is that the optimal concentration to limit the emergence of resistance lies at either end of the feasible window. This relies on the relationship being always unimodal, which is supported by simulations of plausible models. The relationship between the emergence of resistance and drug dose was unimodal in several experimental systems (Bhavnani, Ambrose, Hammel, Rubino, & Drusano, 2016; Firsov et al., 2017; Strukova, Portnoy, Zinner, & Firsov, 2016; Tam, Louie, Deziel, Liu, & Drusano, 2007; VanScoy, McCauley, Bhavnani, Ellis‐Grosse, & Ambrose, 2016). The unimodal (also called “inverted‐U‐shaped” or “hump‐shaped”) relationship remains the paradigm in PK/PD studies (Mouton et al., 2011; Singh & Tam, 2011).

### 3.3 Optimal duration of antibiotic treatment

A large part of the literature is concerned with the optimal antibiotic dose, and fewer theoretical studies have addressed the optimal duration of treatment (but see D'Agata et al., 2008; Geli et al., 2012). The effect of a long versus short duration of treatment may be similar to that of high versus low dose. Actually, some experimental studies find that the area under the curve describing the drug concentration as a function of time (i.e., the product of the mean dose and treatment duration) best predicts the emergence of resistance (Olofsson & Cars, 2007). Longer course duration is associated with a higher frequency of resistance in several experimental studies (Drusano, Liu, Brown, Rice, & Louie, 2009; Martinez, Papich, & Drusano, 2012; Mouton et al., 2011; Nguyen et al., 2014), leading to the suggestion that short antibiotic courses may limit the emergence of resistance at the population level, and studies to determine whether such short course duration would lead to good infection outcomes (Martinez et al., 2012).

### 3.4 Fitting dynamical models to experimental data

The within‐host dynamics of antibiotic resistance is sufficiently well understood to fit explicit dynamical models to experimental data. Jumbe et al. (2003) fitted a dynamical model including the pharmacokinetics of the drug, and the impact of fluoroquinolone treatment on the density of sensitive and resistant strain, in mice colonized by *P. aeruginosa*. They estimated that the growth rate of resistant strains in the absence of treatment was 70% that of the sensitive strain, but the bactericidal activity of fluoroquinolone was eight times smaller on the resistant strain. They validated their model on independent data with different antibiotic doses, successfully predicting that a low dose would encourage the selection of resistance, while a high dose would suppress it. Nguyen et al. (2014) fitted a similar dynamical model to the dynamics of resistance and sensitive *E. coli* strains within the gut of piglets under fluoroquinolone treatment. There, the growth rate of the resistant strain was 14% that of the sensitive strain, and fluoroquinolone was estimated to have no effect at all on the resistant strain.

## 4 CONCLUSION AND PERSPECTIVES

I highlight five important features of models of antibiotic resistance evolution, five insights from models of antibiotic resistance evolution and four perspectives for future work.

### 4.1 Five important features of models of antibiotic resistance evolution

1. The competition between sensitive and resistant strains is key. Some models only consider the resistant strain, implicitly assuming that it does not compete with the sensitive strain. This may be justified by experimental data: for example, one study showed that colonization with a vancomycin sensitive enterococcus strain does not prevent further colonization by a vancomycin‐resistant enterococcus (Huang et al., 2011). However, in general, if two strains differ only in their resistance to antibiotics, it is biologically plausible that they will use the same resources and compete within the host.
2. For many bacterial species, antibiotic treatment is prescribed most often for viral or bacterial infections unlinked with colonization by the focal species. Thus, a large fraction of the exposure to antibiotics and selection for resistance is independent of infection by the focal species.
3. Many bacterial species experience transient (1–2 weeks) and rare (in the order of once per year on average in the community) antibiotic courses in the community. Thus, the “niche” formed by treated individuals for resistant strains is small and transient. A few species causing long infections, such as *M. tuberculosis* or *P. aeruginosa* in cystic fibrosis, may face exceptionally long antibiotic courses.
4. De novo evolution of resistance by point mutation is documented mostly for long duration treatments, presumably because evolution by point mutation is rare for short treatments.
5. Genomic data sets reveal that resistance genes or mutations are confined to clones whose resistance is stable over decades, and that horizontal gene transfers leading to new circulating resistant strains are also rare at epidemiological timescales.

### 4.2 Five insights from models of antibiotic resistance evolution

1. Sensitive and resistant strains are in competition to colonize largely the same hosts. The large overlap in their niches implies competitive exclusion of one or the other strain in most models. There is no one single mechanism explaining the observed long‐term coexistence of sensitive and resistant strains, but several plausible mechanisms such as host population structure and genetic association with other loci could act together to maintain coexistence. More speculatively, resistance genes may have pleiotropic effects beyond their cost that may favour coexistence, or the within‐host environment may present heterogeneities allowing coexistence within hosts.
2. Hospitals have their own dynamics, with a high rate of admission and discharge and high rates of antibiotic treatment. Resistance evolution in hospitals is not unlike adaptation to a “sink” environment, with constant influx of sensitive strains from the “source” community. In these controlled settings, it is possible to explicitly fit dynamical models to data.
3. For bacterial species colonizing humans and farm animals, antibiotic consumption in farm animals could speed up the emergence of new types of resistances—farm animals could act as “incubators” of resistance. However, once a resistance is established, there is little evidence that antibiotic consumption in farm animals influences the frequency of resistance in the human community. More work would be needed to estimate quantitatively the rates of transmission between reservoirs with the help of genomic data.
4. There is no consensus on the best antibiotic prescription strategy to limit resistance in intensive care units or hospitals. Differences between mixing, cycling and reactive cycling strategies are small compared to stochastic effects. Clinical trials are few and have been inconclusive so far.
5. Models of within‐host resistance evolution can be compared with in vitro and in vivo experimental results. The emergence of resistance is favoured at an intermediate antibiotic dose. At a low antibiotic dose, the sensitive strain is unaffected by treatment and competitively suppresses the less fit resistant strain. At a high antibiotic dose, the resistant strain is cleared by antibiotics. This prediction is generally verified experimentally. Dynamical models can successfully be fitted to data in vivo.

### 4.3 Four perspectives for future work

1. We are largely unable to fit dynamical models of resistance evolution to epidemiological data on the frequency of resistance. This means that we do not know what phenomena determine the frequency of resistance and we cannot predict the future evolution of resistance. More realistic model outcomes (in particular, robust coexistence of sensitive and resistant strains) may be obtained with more complex models. The challenge will be to formulate models that remain tractable and understandable, and can be fitted to data.
2. Very few models consider multiple loci under selection (Day & Gandon, 2012). Resistances to several antibiotics depend on multiple genes or loci, but it remains unclear how these resistances are co‐selected to generate multidrug resistance (Lehtinen, Blanquart, Lipsitch, Fraser, & Maela Pneumococcal Collaboration Collaboration, 2017). This is particularly interesting in the light of the observation that multidrug resistance is common, and some plasmids carry multiple resistance genes—so‐called “genetic capitalism.” Moreover, resistance might interact epistatically with other epidemiological traits (such as carriage duration, determined by serotype in *S. pneumoniae*, Lehtinen, Blanquart, Croucher, et al., 2017). As rates of recombination in bacteria are small enough for epistasis to select for combinations of alleles powerfully (Arnold et al., 2018), multilocus models of resistance evolution are an interesting avenue for future research.
3. How resistance evolves within hosts is comparatively better understood (although data on within‐human evolution of resistance are lacking). It would be desirable to develop new models integrating the within‐host dynamics of sensitive and resistant strain at the between‐host level. For example, Webb, D'Agata, Magal, and Ruan (2005) developed a conceptual model linking the within‐host dynamics of sensitive and resistance bacteria to the prevalence in the hospital. This model was extended to account for host immunity and investigate the impact of treatment timing, duration and dose on resistance (D'Agata et al., 2008, 2007) as described above. Other models have taken a very detailed approach (Caudill & Lawson, 2017). Lastly, it has been suggested that more explicitly modelling the within‐host dynamics, and in particular the dual carriage of resistant and sensitive strains, helps maintain between‐host coexistence (Davies, Flasche, Jit, & Atkins, 2017). Pursuing this line of research and more explicitly modelling within‐host dynamics happening on the same timescale as the between‐host dynamics may generate novel insights not captured by the classical compartmental models.
4. Although genomic data are fast accumulating, and the genetic determinants of resistance—the genotype–phenotype map—are very precisely known, no attempt has been made to my knowledge to link dynamical model describing the selection on resistant and sensitive strains to the phylogenetic history of these strains (see Box 2 on phylodynamics).

To conclude, the evolutionary epidemiology of antibiotic resistance is an interesting testing ground for evolutionary theory. It benefits from extensive epidemiological, experimental and genomic data, a well‐developed modelling tradition, and an intuition for what environment selects for antibiotic resistance. Important challenges lie ahead: developing models with predictive ability and understanding what forces maintain diversity despite selection—a particular instance of a major question in evolutionary biology.

## CONFLICT OF INTEREST

None declared.
