# Supplementary material for: Evolutionary epidemiology models to predict the dynamics of antibiotic resistance
Source: Evol Appl. 2019 Jan 21;12(3):365–83. doi: 10.1111/eva.12753 (PMC6383707; doi:10.1111/eva.12753)
Supplement: Supplementary file 1 [file EVA-12-365-s001.pdf]

Supplementary information for "Evolutionary epidemiology models to predict the dynamics of antibiotic resistance"

This text describes in details the three evolutionary epidemiology models presented as examples in the main text.

Between-host model of resistance evolution (fig. 2)

A model of resistance evolution is pictured on fig. 2. This model has six variables:

- Uncolonised (susceptible) untreated inviduals X^U
- Uncolonised treated individuals X^T
- Individuals colonised by the sensitive strain, untreated, S^U
- Individuals colonised by the sensitive strain, treated, S^T
- Individuals colonised by the resistant strain, untreated, R^U
- Individuals colonised by the resistant strain, treated, R^T

The dynamics of these variables is given by the following ordinary differential equations (ODE) model:

$$\dot{X^U} = -\beta_S(S^U + S^T)X^U - \beta_R(R^U + R^T)X^U + u_S S^U + u_R R^U - \tau X^U + \omega X^T$$

$$\dot{X^T} = -\beta_S(S^U + S^T)X^T - \beta_R(R^U + R^T)X^T + u_S S^T + u_R R^T + a_S S^T + a_R R^T + \tau X^U - \omega X^T$$

$$\dot{S^U} = \beta_S(S^U + S^T)X^U - u_S S^U - \tau^C S^U + \omega S^T - \mu S^U + \nu R^U$$

$$\dot{S^T} = \beta_S(S^U + S^T)X^T - u_S S^T - a_S S^T + \tau^C S^U - \omega S^T - \mu^T S^T + \nu^T R^T$$

$$\dot{R^U} = \beta_R(R^U + R^T)X^U - u_R R^U - \tau R^U + \omega R^T + \mu S^U - \nu R^U$$

$$\dot{R^T} = \beta_R(R^U + R^T)X^T - u_R R^T - a_R R^T + \tau R^U - \omega R^T + \mu^T S^T - \nu^T R^T$$

The terms in blue represent transmission, happening at rate β_S et β_R for the sensitive and resistant strains. The terms in green represent natural clearance at rates u_S and u_R for the sensitive and resistant strains. The terms in red represent clearance by antibiotic treatment at rates a_S and a_R. The terms in purple represents treatment start at rate τ (τ^C for colonised individuals), and stops at rate ω (the average duration of treatment is $1/\omega$). Lastly, the terms in gray represent the evolution of resistance *de novo* within treated and untreated hosts (at rates μ^T and μ) and the reversion of resistance (at rates ν^T and ν).

In this model, the total host population size is constant (the sum of all variables time derivatives is 0).

Hospital model of resistance evolution (fig. 3)

This model is an extension of the between-host model of evolution to two antibiotics A and B and two corresponding resistances A and B. Thus, the model includes 12 variables corresponding to uncolonised hosts, hosts colonised by the sensitive strain, hosts colonised by the A-resistant strain, and hosts colonised by the B-resistant strain, times three treatment status: untreated, treated by the antibiotic A, treated by the antibiotic B. The evolution of dual resistance to both antibotics A and B is not modelled.

The model describes the dynamics of resistance in the hospital. In addition to extending the between-host model to two resistances, the model includes an influx of individuals from the

community and an efflux from the hospital. Efflux of all hosts happens at a rate e (regardless of their status). The total influx exactly balances the total efflux, such that the total host population remains constant in the hospital.

The model compares different treatment strategies. Specifically, given a fixed total treatment rate τ_{tot}, it compares:

- A single antibiotic strategy whereby $\tau_A = \tau_{tot}$ and $\tau_B = 0$ ("one antibiotic")
- A 50%-50% mixing strategy whereby $\tau_A = \tau_B = \tau_{tot}/2$ ("50-50 mixing")
- A sine wave cycling strategy whereby $\tau_A = \frac{\tau_{tot}}{2}\left(1 + \sin\left[\frac{2\pi t}{T}\right]\right)$ and $\tau_B = \frac{\tau_{tot}}{2}\left(1 - \sin\left[\frac{2\pi t}{T}\right]\right)$ ("cycling").

In addition, we compare these strategies with a strategy where one antibiotic only is used and the transmission rate is reduced by 50% ("reduced transmission").

Within-host model of resistance evolution (fig. 4)

The model (fig. 4) describes the within-host antibiotic concentration C, and the density of sensitive and resistant strains S and R, with the following ordinary differential equations:

$$\frac{dC}{dt} = T - k_e C$$

$$\frac{dS}{dt} = \alpha_S\left(1 - \frac{R+S}{N_{max}}\right)S + g_S - k_T\,S - \delta_{max}\frac{C}{C + C_S^{50}}\,S$$

$$\frac{dR}{dt} = \alpha_R\left(1 - \frac{R+S}{N_{max}}\right)R + g_R - k_T\,R - \delta_{max}\frac{C}{C + C_R^{50}}R$$

In the first equation, which describes the dynamics of antibiotic concentration, T is a positive constant when the individual is treated, and *0* when the individual is untreated. The term in $-k_e C$ quantifies the efflux or elimination of the antibiotic.

The different terms of the dynamics of bacterial density are indicated with colours (unrelated to the colour code used for the other models). The terms in blue correspond the within-host growth of the bacteria, at rates α_S and α_R for the sensitive and resistant strains. Growth is density-dependent and the density at which growth cancels is N_{max}. The terms in green represent the influx of sensitive and resistant strains through successful transmission, at rates g_S and g_R. The terms in red represent the efflux of bacteria at rate k_T. The term in purple represents the antibiotic killing action at a rate that depends on the antibiotic concentration. δ_{max} is the maximum rate. C_S^{50} and C_R^{50} are the antibiotic concentration where the killing action is half its maximum value, for the sensitive and resistant strains respectively.
